# Effects of Heat Stress on Heart Rate Variability in Free-Moving Sheep and Goats Assessed With Correction for Physical Activity

**DOI:** 10.3389/fvets.2021.658763

**Published:** 2021-06-01

**Authors:** Kaho Kitajima, Kazato Oishi, Masafumi Miwa, Hiroki Anzai, Akira Setoguchi, Yudai Yasunaka, Yukiko Himeno, Hajime Kumagai, Hiroyuki Hirooka

**Affiliations:** ^1^Laboratory of Animal Husbandry Resources, Division of Applied Biosciences, Graduate School of Agriculture, Kyoto University, Kyoto, Japan; ^2^Division of Grassland Farming, Institute of Livestock and Grassland Science, National Agriculture and Food Research Organization, Tochigi, Japan; ^3^Department of Animal and Grassland Sciences, Faculty of Agriculture, University of Miyazaki, Miyazaki, Japan; ^4^Department of Bioinformatics, College of Life Sciences, Ritsumeikan University, Shiga, Japan

**Keywords:** physical activity, non-linear analysis, heat stress, heart rate variability, dynamic body acceleration

## Abstract

Heart rate variability (HRV) is the heart beat-to-beat variation under control of the cardiovascular function of animals. Under stressed conditions, cardiac activity is generally regulated with an upregulated sympathetic tone and withdrawal of vagal tone; thus, HRV monitoring can be a non-invasive technique to assess stress level in animals especially related to animal welfare. Among several stress-induced factors, heat stress is one of the most serious causes of physiological damage to animals. The aim of this study was to assess the effects of heat stress on HRV in small ruminants under free-moving conditions. In three experimental periods (June, August, and October), inter-beat intervals in sheep and goats (three for each) in two consecutive days were measured. HRV parameters were calculated from the inter-beat interval data by three types of analyses: time domain, frequency domain, and non-linear analyses. The temperature–humidity index (THI) was used as an indicator of heat stress, and vectorial dynamic body acceleration (VeDBA) was calculated to quantify the physical activity of the animals tested. First, we investigated correlations of THI and VeDBA with HRV parameters; subsequently, THI was divided into five categories according to the values obtained (≤ 65, 65–70, 70–75, 75–80, and >80), and the effects of the THI categories on HRV parameters were investigated with and without correcting for the effects of physical activity based on the VeDBA. The results indicated that HRV significantly decreased with increasing THI and VeDBA. For non-linear HRV parameters that were corrected for the effects of physical activity, it was suggested that there would be a threshold of THI around 80 that strongly affected HRV; high heat stress can affect the autonomic balance of animals non-linearly by inducing the sympathetic nervous system. In conclusion, to assess psychophysiological conditions of unrestrained animals by HRV analysis, the confounding effect of physical activity on HRV should be minimized for a more precise interpretation of the results.

## Introduction

Heart rate variability (HRV) has been used as a sensitive indicator of the functional regulatory characteristics of the autonomic nervous system (1, 2). Inter-beat interval fluctuation expressed as HRV reflects the sympathetic and parasympathetic activity of the autonomic nervous system, and healthy cardiac function is characterized by irregular time intervals between consecutive heart beats (2). A decrease in HRV can be caused by an increase in sympathetic activity and/or withdrawal of parasympathetic activity, and HRV is a particularly good indicator for the non-invasive assessment of autonomic nervous system activity in response to various internal and external stressors.

Several studies have investigated HRV in animals: farm animals (2), companion animals (3, 4), monkeys (5), seabirds (6), etc. In particular, monitoring HRV in animals has recently gained attention as a non-invasive technique of assessing stress levels of animals related to animal welfare (7). For example, numerous studies have investigated internal and external psychological and physiological factors that affect the HRV of dairy cattle: temperament and reactivity to humans (8–10), bell noise (11), palpation (12, 13), insect harassment (14), seasonality (15), milking system (16, 17), pregnancy and calving (18–21), and diseases such as lameness (22), diarrhea (14), and bovine spongiform encephalopathy (23). However, physical activity (movement) influences HRV, and it can cloud the regulation linked to cognitive, emotional, social, and health process (24, 25). In fact, HRV is greatly affected by the behavior of animals, and some studies have focused on the effects of behavior on HRV, such as body posture (9), step behavior (17), and behavioral types (26). When the effects of psychophysiological changes on HRV of animals have to be evaluated, particularly under free-moving conditions, the quantified physical activity level of the target animals should also be considered as a key element that influences HRV (27).

Heat stress is a serious cause of physiological damage on animals and consequently affects their production (28–31). Many previous studies on heat stress for animals have focused on its negative effects on productivity (32–36) and physiological reactions related to hormonal and immune responses (37–40). For example, in dairy cattle, as milk production increases, metabolic heat production rises with the metabolism of large amounts of nutrients, which makes the high-producing cows more vulnerable to high ambient temperatures and humidity than animals that are less active metabolically (28). Also, heat stress compromises productivity in small ruminants, increasing maintenance energy requirement (41). For investigating such effects of heat stress on animals, thermoregulatory function traits such as respiration rate, rectal temperature, and heart rate were widely evaluated as physiological indicators of heat stress (34). However, although the effects of shading on HRV in cattle under extreme heat loads have recently been investigated (42, 43), no studies have analyzed the effects of heat stress on the autonomic nervous system of animals in detail using a variety of HRV analyses, in particular by considering the effect of physical activity. Regarding the variety of HRV analyses, not only conventional time domain and frequency domain indices but also non-linear indices have been used as reliable markers of sympathetic and vagal activation in HRV analysis (1). Quantifications in different domains of HRV analysis can focus on different characteristics of HRV: quantity of variance (time domain), periodic processes due to autonomic regulation (frequency domain), and chaotic phenomena in the regulation of cardiac activity (non-linear); and HRV parameters derived from such different domains can complement each other (16). Therefore, evaluation of different HRV parameters can be adequate to judge different quantitative and qualitative stress loads in animals (14). In order to successfully manage healthy animals, HRV assessment using a variety of parameters in multiple domains appears to be required, which provides quantitative and objective assessments of the effect of heat stress on the autonomic nervous system activity under free-moving conditions.

The aim of the present study was to assess the effects of heat stress on HRV in small ruminants using three types of analyses: time domain, frequency domain, and non-linear analyses. To elucidate the physiological interrelationships between heat stress and HRV under free-moving conditions, the effects of heat stress on HRV were evaluated with and without correcting for the confounding effect of physical activity on HRV by simultaneously measuring the dynamic body acceleration of the animals.

## Materials and Methods

### Animals and Experimental Periods

All of the data were obtained from experiments conducted at Kyoto University, Japan (35°02′N, 135°47′E). Three castrated Corriedale sheep that are 2.5 years old and three castrated Japanese Saanen goats that are 9 years old were used. The experiments were conducted in three periods: June (early summer), August (midsummer), and October (early autumn) in 2015. Data collection was carried out for all sheep and goats simultaneously, and the data were collected consecutively for about 2 days per animal in each period. The body weights (kg) of the animals were measured on the first day of each respective period: 34.3 ± 4.9, 34.9 ± 5.4, and 35.0 ± 2.5 kg for the sheep and 73.9 ± 3.9, 76.1 ± 2.2, and 72.8 ± 4.4 kg for the goats. The sheep and goats were separately managed into two pens (each species per pen: ~10 m^2^ per pen) in an open-sided, metal-roofed animal shelter. The sheep was shorn in the spring following conventional management. The animals were fed twice a day (9:30 and 15:30), and the amount of feed offered was 2% of their body weight per day (Italian ryegrass: alfalfa hay cube: concentrate = 10:3:3) as the amount required for maintenance. Mineral blocks and water were freely available. All of the animal experiments were approved by the Animal Experiment Committee of Kyoto University (permit number: 27-56). All of the procedures for equipping the animals with data loggers were performed as quickly as possible to minimize the animals' discomfort. All animals were housed for use in future researches.

### Data Collection

**Figure 1:**
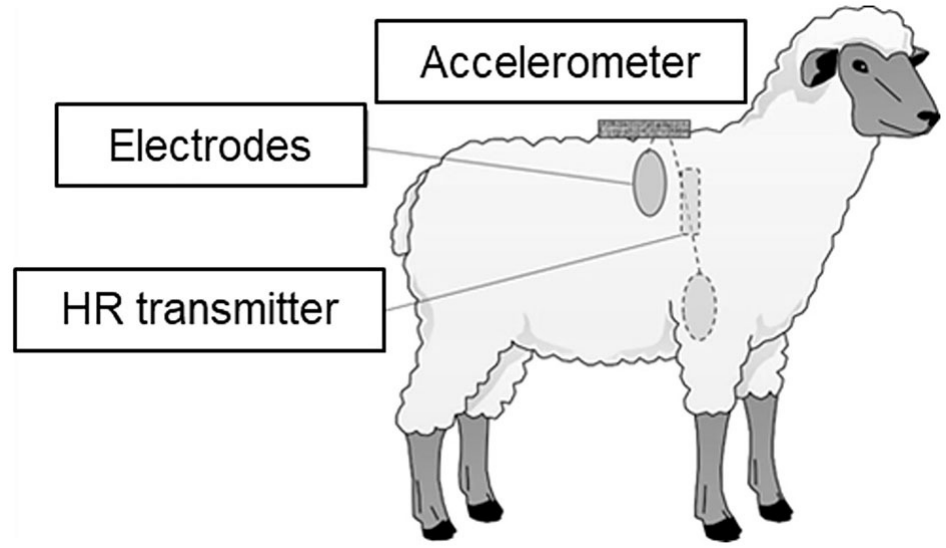
Positions of heart rate (HR) transmitter, electrodes for the HR monitor, and the accelerometer.

Inter-beat interval data (ms) were obtained using heart rate monitors (RS800CX and H2 heart rate sensors, Polar Electro Ltd., Finland). Each heart rate monitor consisted of a transmitter with two electrodes and a logger. The electrodes were placed on the animal's right shoulder and left anterior thorax and attached with a homemade chest belt (44). The electrode sites of animals were roughly shaved and covered with a conductive gel to optimize electrode contact. In addition, three-dimensional accelerations were simultaneously recorded using acceleration data loggers (USB Accelerometer X6-1A and X6-2A, Gulf Coast Data Concepts, Waveland, MS, USA). The acceleration logger was attached to the back of the animals when the heart rate monitor was attached. Accelerations were recorded at 10 Hz, with a 16-bit resolution. The positions of the heart rate monitor and acceleration logger are shown in Figure 1.

During each experiment, ambient temperature and relative humidity per minute were measured using a thermo-hydro data logger (TR-72wf-H, T&D Corporation, Japan). The logger was placed in the animal shelter.

In addition, respiration rate and rectal temperature, traditional physiological indices, were measured on the last day of each experimental period. Respiration rate was measured visually by counting thoracoabdominal movements for 30 s twice by each of three observers on the last day of each experimental period at a distance of ~1 m between animals and observers. Then, the doubled value of the average of six counting values by the three observers was taken as the respiration rate per minute for each animal in each period. The observation was carried out for the target animals in standing position. Rectal temperature was measured once for each animal at the end of each experiment (before evening feeding) by a digital thermometer (MC-170, Omron Healthcare Co., Ltd., Japan).

### Temperature–Humidity Index

The effect of heat is aggravated when heat stress is accompanied with high ambient humidity (34). Therefore, the THI was used as an indicator of heat stress in the present study, which was calculated from the average values of ambient temperature and relative humidity in 5 min using the following equation (45):

$$\begin{array}{l} THI=0.8\times temperature+\left(humidity/100\right)\\ \;\times \left(temperature-14.4\right)+46.4. \end{array}$$

### Effect of Physical Activity on HRV

A change in physical activity levels is thought to be one of the main modulators of HRV (46). Because cardiac activity is affected by variations in the respiration rate via sympathetic and parasympathetic nerves (47), HRV is greatly influenced by the change in respiration caused by physical activity. Therefore, to improve the precision of HRV evaluation under free-moving conditions, the effect of physical activity on HRV should be considered. One of the possible methods to consider the effect is to delete the sections of the heart inter-beat interval data with excessive movement before the HRV analysis (48–50), but there have been no general ways for separating the effect of changes in quantified physical activity levels on HRV from the influence of other regulatory processes (24). Therefore, in this study, the vectorial dynamic body acceleration (VeDBA), which can be a proxy for the activity-specific energy requirements of animals (25, 51, 52), was used to remove the confounding effect of physical activity on HRV based on the method by Oishi et al. (27). To calculate VeDBA, triaxial acceleration values obtained by the acceleration loggers were used in the following equation:

$$\begin{array}{l} VeDBA=\sqrt{\left(D_{x}^{2}+D_{y}^{2}+D_{z}^{2}\right)}, \end{array}$$

where *A*_*x*_, *A*_*y*_, and *A*_*z*_ are triaxial dynamic body accelerations calculated by subtracting static accelerations from raw accelerations (53). Initially, the VeDBA values were transformed into natural logarithms for normalization, which were then averaged in 5 min to correspond with the respective HRV data and used for the analysis.

### HRV Analysis

Kubios HRV 2.2 software (Kubios Ltd., Finland) (54) was used to calculate HRV parameters from inter-beat interval data. All of the HRV parameters for both animal species were calculated by 5-min windows according to the Task Force of the European Society of Cardiology, North American Society of Pacing and Electrophysiology (55). A total of 9,963 adjacent 5-min windows were obtained for the analysis. Artifacts were removed from the data using a threshold-based artifact correction algorithm built-in in this software with a medium correction level (54). Moreover, data that included 5-min-averaged inter-beat interval values outside the 3-sigma range of individual datasets were also removed. The proportion of data removed was 1.13%. A total of 9,850 5-min intervals, with an average of 547.2 intervals (45.6 h) per animal in each period, were included in the analysis. For each animal, the intervals were almost evenly distributed across each period.

In the present study, the mean heart rate per 5 min (HR) and the following six HRV parameters were calculated using three domain analyses: time domain parameters (SDNN and RMSSD) from time domain analysis, frequency domain parameters (HF and LF/HF) from frequency domain analysis, and non-linear domain parameters (L_max_ and %DET) from non-linear domain analysis. These parameters are defined in Table 1.

**Table 1:** Definitions of HRV parameters.

**Parameter^**a**^**	**Definition (unit)**
**Time domain**	
SDNN	Standard deviation of inter-beat intervals (ms)
RMSSD	Square root of the mean squared differences of successive inter-beat intervals (ms)
**Frequency domain**	
HF	Normalized power of the high-frequency band (n.u.): 0.2–0.4 Hz
LF/HF	Ratio of the normalized power of the low-frequency (LF) band (n.u.) (0.04–0.2 Hz) to HF
**Non-linear**	
L_max_	The length of the longest line of recurrent points (beats)
%DET	Percentage of recurrent points that appear in sequence (%)

*HRV, heart rate variability.*

a *In general, animals with low HRV exhibit high sympathetic activity, which can be associated with an increased risk of stress. SDNN, RMSSD, and HF are positively related to HRV, whereas the other parameters are negatively related to HRV*.

Time domain analysis is the simplest form of HRV analysis. Time domain parameters reflect various aspects of the statistical variability of inter-beat intervals and are often used to interpret HRV characteristics. The standard deviation of inter-beat intervals (SDNN) is a good predictor of overall variability influenced by both sympathetic and parasympathetic activity, and the root mean square of successive inter-beat interval differences (RMSSD) is the primary time domain measure used to estimate the high-frequency beat-to-beat variation that represents vagal tone activity (2).

Frequency domain analysis is the procedure of decomposing a waveform of inter-beat intervals by range of the frequency using fast Fourier transformation (FFT). In this analysis, the waveform of inter-beat intervals is regarded as a synthesized waveform, and the power spectra of the waveform are calculated to estimate the HRV. An increase in high-frequency (HF) components is generally caused by increasing HRV, and HF has been used to describe the function of vagal tone (56). In addition, the ratio of the power spectra of low-frequency (LF) components to HF (LF/HF) is utilized for describing the sympathovagal balance of the autonomic nervous system (57). The ranges of the HF and LF components for the tested animals were set to be 0.20–0.40 and 0.04–0.20 Hz, respectively, as recommended by von Borell et al. (2).

Non-linear domain analysis elucidates the chaotic behavior of HRV using non-linearity indicators. A great deal of information can be extracted from physiological signals by describing their dynamic behavior, and non-linearity is the representative indicator of such complex dynamical systems (58). Recurrence quantification analysis (RQA) is a method of non-linear HRV analysis that was developed by Eckmann et al. (59), which has been used to detect hidden and complex characteristics of HRV (60). In the present study, the length of the longest line of recurrence points (L_max_) and the percentage of determinism (%DET) were used as the quantitative parameters of RQA. These parameters reflect the richness of the deterministic structure of inter-beat intervals in a time series, and an increase in these parameters indicates a decrease in HRV (61). The evaluation of non-linear parameters with time domain and frequency domain parameters is a useful method of analyzing HRV for the accurate interpretation of the autonomic nervous system function (14, 16).

### Statistical Analysis

Ambient temperature, relative humidity, and THI were analyzed by one-way analysis of variance (ANOVA), with period (June, August, and October) included as a fixed effect. Respiration rate and rectal temperature were analyzed by two-way ANOVA, with period (June, August, and October) and species (sheep and goats) included as fixed effects.

Regarding HRV, Pearson's correlation coefficients of mean HR and HRV parameters with THI and VeDBA were firstly calculated. In addition, the THI was divided into five categories (≤ 65, 65–70, 70–75, 75–80, and >80), and the effects of the THI category on mean HR and HRV parameters were analyzed using the following linear mixed model:

$$\begin{array}{l} Y_{ijkl}=\mu +catTHI_{i}+Species_{j}+Animal_{k\left(j\right)}+e_{ijkl}, \end{array}$$

where *Y*_*ijkl*_ is the value of the mean HR and HRV parameters (SDNN, RMSSD, HF, LF/HF, L_max_, and %DET) per 5 min, μ is the overall mean, *catTHI*_*i*_ (*i* = 1–5) is the fixed effect of the THI category, *Species*_*j*_ (*j* = 1 or 2) is the fixed effect of species, *Animal*_*k*__(j)_ (*k* = 1–6) is the random effect of individual animals nested within species, and *e*_*ijkl*_ is the error. Furthermore, based on the method by Oishi et al. (27), the above statistical model was transformed into the following model in order to include the effect of quantified physical activity on mean HR and HRV parameters:

$$\begin{array}{l} Y_{ijkl}=\mu +catTHI_{i}+Species_{j}+Animal_{k\left(j\right)}+{\beta \left(VeDBA\right)}_{ijkl}\\ \;+e_{ijkl}, \end{array}$$

where β (*VeDBA*)_*ijkl*_ is the covariate effect of VeDBA per 5 min.

Differences were analyzed using the least squares means with the Tukey–Kramer *post-hoc* test (62) and were considered significant at *P* < 0.05. Correlation coefficients were calculated using PROC CORR, and the other analyses were performed using PROC MIXED in SAS 9.3 (SAS Institute) (63).

## Results

### Respiration Rate and Rectal Temperature and Changes in Environmental Conditions

The least square means of ambient temperature, relative humidity, and THI during each experimental period are shown in Table 2. The ambient temperature and THI in August were the highest, and those in October were the lowest (*P* < 0.05). The relative humidity in June was higher than that in August or October (*P* < 0.05).

**Table 2:** Ambient temperature, relative humidity, and THI during the experimental periods.

**Index**	**June**	**August**	**October**
Temperature (°C)	22.6 ± 0.05^b^ (18.5–31.7)	29.1 ± 0.06^a^ (24.7–36.4)	18.4 ± 0.06^c^ (13.1–26.1)
Humidity (%)	71.7 ± 0.19^a^ (37.2–83.6)	58.9 ± 0.23^b^ (36.0–74.3)	58.8 ± 0.22^b^ (30.5–82.0)
THI	70.1 ± 0.06^b^ (64.5–78.3)	78.0 ± 0.07^a^ (73.1–83.7)	63.0 ± 0.06^c^ (55.8–70.8)

*THI, temperature–humidity index. Values are least squares means ± standard errors and ranges. Different letters indicate a significant difference between periods (P < 0.05)*.

The least square means of respiration rate and rectal temperature are shown in Table 3. The respiration rate in August was the highest (*P* < 0.05), and the respiration rate in sheep tended to be higher than that in goats (*P* = 0.053). The rectal temperature of sheep was higher than that of goats (*P* < 0.05), but no significant effect of period was found.

**Table 3:** Respiration rates and rectal temperatures of the tested animals.

**Species**	**Month**	**Respiration rate (/min)**	**Rectal temperature (^**°**^C)**
Goat	June	14.57	39.10
	August	57.63	39.23
	October	16.93	38.60
Sheep	June	39.10	39.47
	August	84.53	39.70
	October	41.17	39.23
SEM		14.37	0.26
Effect of species		*P* = 0.053 (Goat < Sheep)	*P* < 0.05 (Goat < Sheep)
Effect of period		*P* < 0.05 (June and October < August)	n.s.

*Values are least squares means. No significant interaction effects were found. SEM, standard error of the mean; n.s., not significant*.

### Effects of Heat Stress on HRV

Pearson's correlation coefficients between HRV parameters and THI and between HRV parameters and VeDBA are shown in Table 4. Both the THI and VeDBA were significantly correlated with the mean HR and all HRV parameters (*P* < 0.05). From the results of the coefficients, both the THI and VeDBA were negatively correlated with HRV, i.e., positively correlated with LF/HF and the non-linear parameters and negatively correlated with the other HRV parameters. For most of the HRV parameters, HRV was more strongly correlated with VeDBA than with THI.

**Table 4:** Pearson's correlation coefficients of mean HR and HRV parameters with THI and VeDBA.

**Index**	**Mean HR**	**SDNN**	**RMSSD**	**HF**	**LF/HF**	**L_**max**_**	**%DET**
THI	0.352	−0.120	−0.235	−0.211	0.190	0.195	0.179
VeDBA	0.514	0.062	−0.274	−0.414	0.328	0.460	0.449

*All coefficients are significant at P < 0.05.*

*THI, temperature–humidity index; VeDBA, natural logarithmically transformed vectorial dynamic body acceleration (g); HR, heart rate (bpm); HRV, heart rate variability; SDNN, standard deviation of inter-beat intervals (ms); RMSSD, square root of the mean squared differences of successive inter-beat intervals (ms); HF, normalized power of the high-frequency band (n.u.); LF/HF, ratio of the normalized power of the low-frequency (LF) band to HF; L_max_, the length of the longest line of recurrent points (beats); %DET, percentage of recurrent points that appear in sequence (%)*.

**Figure 2:**
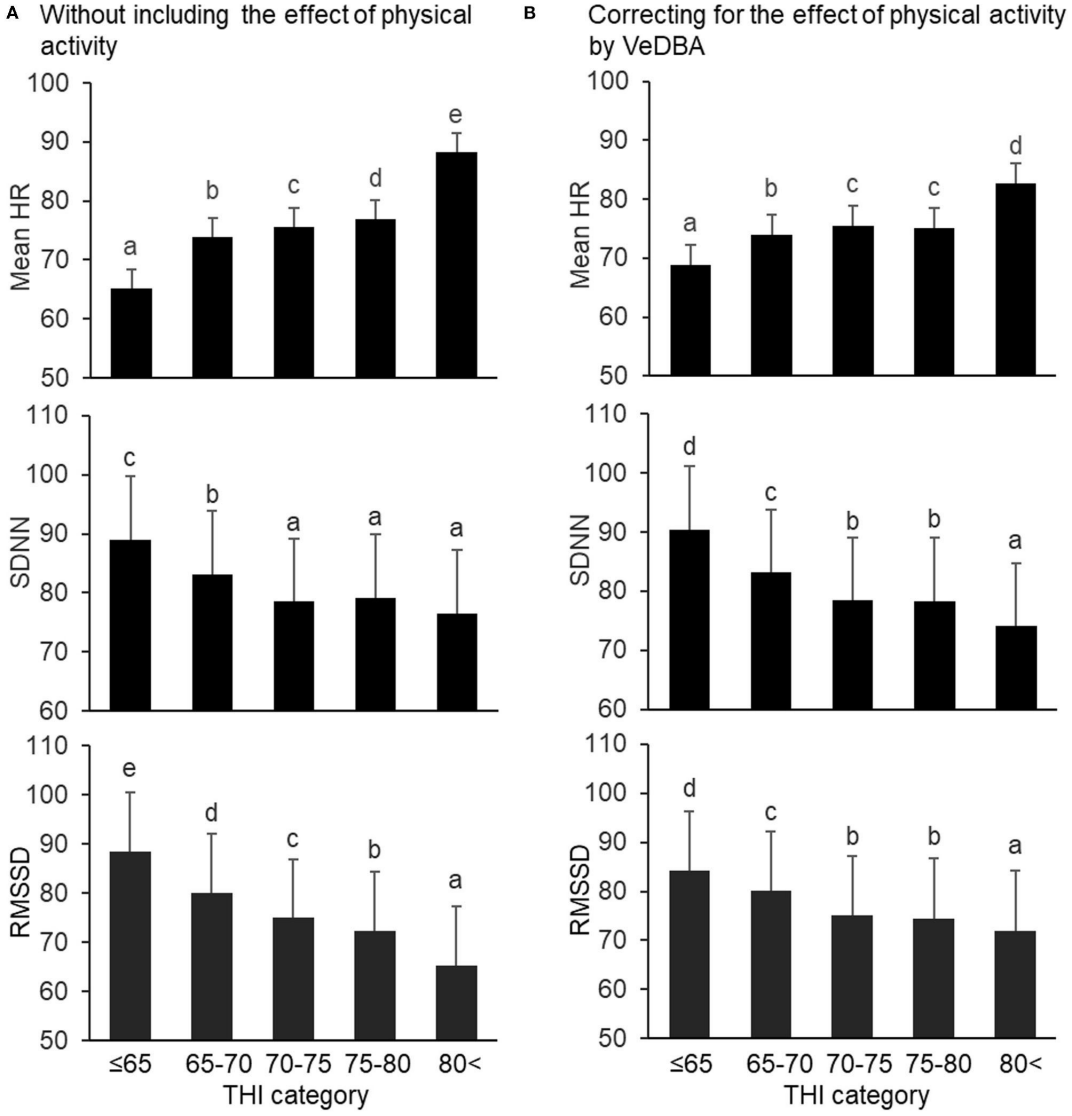
Changes in mean HR and time domain HRV parameters (SDNN and RMSSD) by THI category, **(A)** without and **(B)** with the effect of physical activity quantified by VeDBA. The bars represent least square means, and error bars show standard errors. Values with different letters differ significantly (*P* < 0.05). HR, heart rate (bpm); HRV, heart rate variability; SDNN, standard deviation of inter-beat intervals (ms); RMSSD, square root of the mean squared differences of successive inter-beat intervals (ms); THI, temperature–humidity index; VeDBA, natural logarithmically transformed vectorial dynamic body acceleration (g).

**Figure 3:**
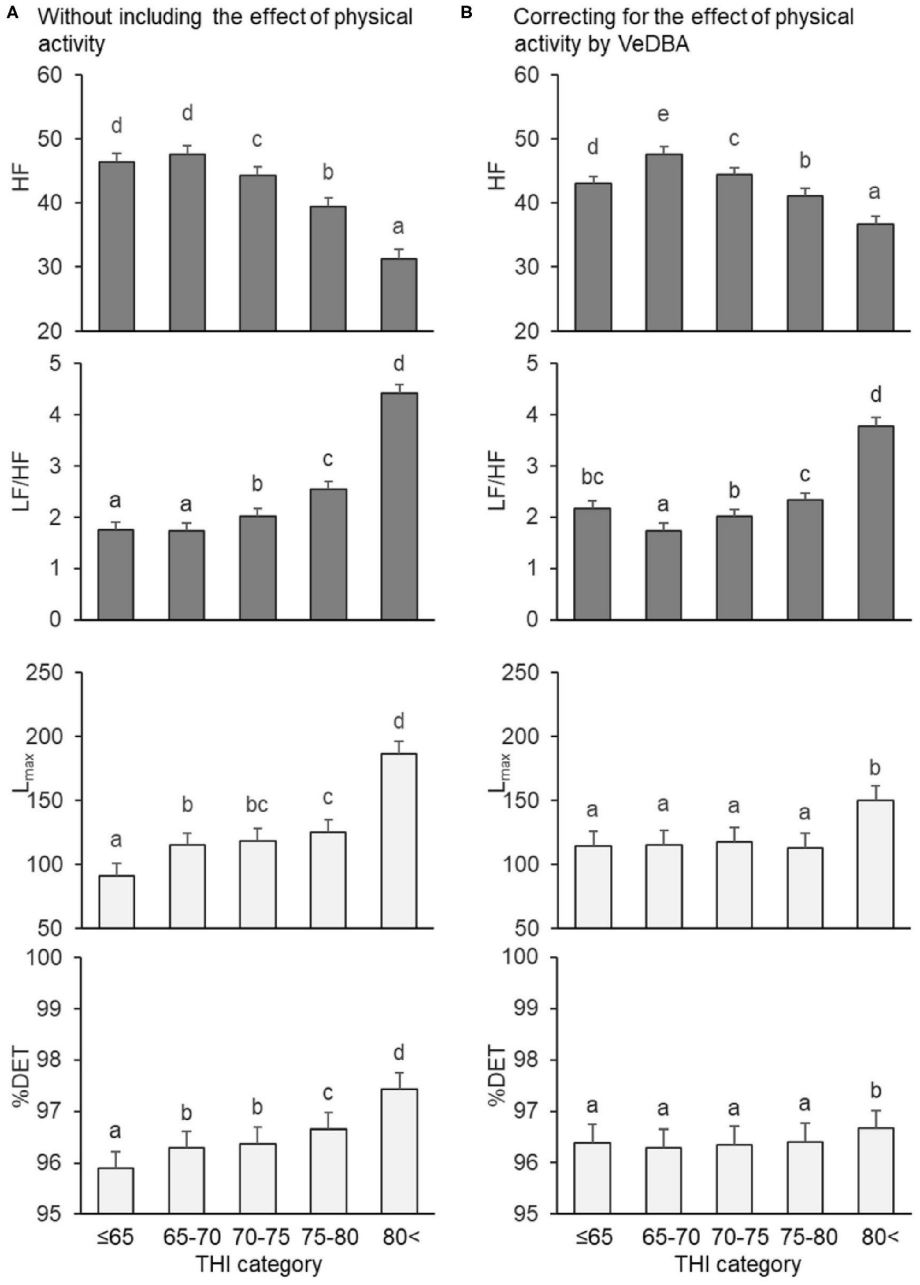
Changes in frequency and non-linear domain parameters by THI category, **(A)** without and **(B)** with the effect of physical activity quantified by VeDBA. HF and LF/HF are frequency domain parameters (gray bars), and L_max_ and %DET are non-linear parameters (white bars). The bars represent least square means, and error bars show standard errors. Values with different letters differ significantly (*P* < 0.05). HRV, heart rate variability; HF, normalized power of the high-frequency band (n.u.); LF/HF, ratio of the normalized power of the low-frequency (LF) band to HF; L_max_, the length of the longest line of recurrent points (beats); %DET, percentage of recurrent points that appear in sequence (%); THI, temperature–humidity index; VeDBA, natural logarithmically transformed vectorial dynamic body acceleration (g).

Results of the changes in the mean HR and HRV parameters in the THI categories by analyzing the models with and without the effect of VeDBA are illustrated in Figure 2 (mean HR and time domain parameters) and Figure 3 (frequency domain and non-linear parameters). Regardless of whether the effect of physical activity was included in the model, the fixed effect of species was not significant for the mean HR and HRV parameters, except for the two frequency domain parameters; HF in goats was significantly higher than that in sheep, and LF/HF in goats was significantly lower than that in sheep (*P* < 0.05). The fixed effect of the THI category was significant for the mean HR and all HRV parameters (*P* < 0.05). When the effect of physical activity was included, the covariate of VeDBA was significant for the mean HR and all HRV parameters (*P* < 0.05).

Regarding the mean HR and time domain parameters, when the effect of physical activity was not included, the mean HR gradually increased when the THI was ≤ 65 to >80, the SDNN decreased when the THI was ≤ 65 to 70–75 (but did not significantly change from 70–75 to >80), and the RMSSD decreased step by step from THI ≤ 65 to >80 (Figure 2A). Similar results were obtained when the effect of physical activity was included, although differences between 70–75 and 75–80 in mean HR, SDNN, and RMSSD were not significant (Figure 2B). Regarding the frequency domain parameters, when the effect of physical activity was not included, HF decreased and LF/HF increased from 65–70 to >80 (Figure 3A). However, when including VeDBA, HF and LF/HF had maximum and minimum values, respectively, in 65–70 (Figure 3B). With regard to the non-linear HRV parameters, the two non-linear parameters increased from THI ≤ 65 to 65–70, slightly changed from 65–70 to 75–80, and increased again from 75–80 to >80 (Figure 3A). Surprisingly, when the effect of physical activity was included, the non-linear HRV parameters only increased from 75–80 to >80 (Figure 3B), indicating that there was a threshold level between the physiological states of the two THI categories.

## Discussion

Monitoring HRV has been used as a non-invasive method of investigating characteristics of the autonomic nervous system. In the present study, HRV was characterized using three domain parameters in order to reveal the general features of HRV in ruminants under heat-stress conditions.

### Correlations of Heat Stress and Physical Activity With HRV

The two time domain HRV parameters (SDNN and RMSSD) were significantly, negatively correlated with the THI (Table 4). As for the frequency domain parameters, HF was negatively correlated with the THI, whereas LF/HF was positively correlated with it. Therefore, HRV parameters which represent vagal tone function in the two domains (RMSSD and HF) decreased under high-THI conditions, which might be in agreement with the previous study (43) showing that RMSSD of calves decreased under highly heat-stressed daytime. As for the non-linear parameters, L_max_ and %DET were significantly, positively correlated with the THI, which reflected a change toward a decrease in HRV by increasing sympathetic nervous system activity and a more periodic heart rate under stress. Hence, all of the HRV parameters showed that HRV decreased with increasing THI. However, it is noticed that the correlation between THI and HRV was significant but considerably weak, which might be due to the existence of thermoneutral range for tested animals during the experimental periods. In addition, despite the fact that the animals were housed in an animal shelter and their physical activity levels were low, VeDBA was significantly correlated with all HRV parameters (Table 4), which was in accordance with our previous study (27). Furthermore, the absolute values of the correlation coefficients between VeDBA and the HRV parameters were mostly higher than those between the THI and the HRV parameters. These results suggest that animals' physical activity influences HRV and that the relationship between physical activity and HRV may be stronger than that between the THI and HRV. Therefore, we can conclude that correcting for the confounding effect of physical activity is necessary to evaluate the effect of heat stress on HRV more precisely.

### Effects of Heat Stress and Physical Activity on the Three Domains of HRV Parameters

It was supposed that heat stress can interfere non-linearly with the physiological function of animals, although a weak linear correlation between the THI and HRV was found. In the present study, therefore, the THI was divided into five categories (levels) in the statistical model, and the effect of the THI categories on HRV was analyzed with and without correcting for the effect of physical activity (Figures 2, 3). First, regardless of whether VeDBA was included as a covariate in the statistical models, the effect of species was significant only for HF and LF/HF. Frequency domain parameters can be modified when the autonomic nervous system responds to changes in the respiration rate (64). In the present study, the respiration rate of sheep tended to be higher than that of goats (Table 3). Machando et al. (65) suggested a physiological susceptibility to heat in sheep with higher respiration rates when compared with goats. However, Johnson (66) reported that sheep and goats showed similar changes in respiration rate when the animals were shorn to the same hair length before the experiment. Hence, the result of differences in respiration rate in the present study might be due the effect of the regrown hair of sheep during the three experimental periods. Besides, the younger age and smaller body weight of sheep compared with goats also might be causes of this result. Thus, with the inclusion of such several differences between the two small ruminant species, the difference in respiration rate between the species was expressed as the effect of species on the frequency domain parameters of HRV. As for the effect of THI categories on the HRV parameters, when the effect of physical activity on HRV was not included, most of the HRV parameters showed that HRV decreased with increasing THI. However, when the effect of physical activity on HRV was included, we found specific changes with changes in THI category, which highlighted the characteristics of the three domains of analysis.

The HRV parameters in the time domain analysis (SDNN and RMSSD) decreased with increases in the THI categories in both analyses, with and without the inclusion of VeDBA. This finding is in accordance with those from our previous study, indicating that these time domain parameters do not strongly correspond with short-term changes in physical activity (27). In contrast, changes in the frequency domain parameters (HF and LF/HF) differed by the inclusion of the effect of physical activity; they formed curved patterns. It is possible that changes in respiration rate caused by an increase in THI affected the frequency domain parameters, even after correcting for the effect of physical activity. As already indicated, frequency domain HRV parameters can be strongly affected by respiration rate, and it is therefore crucial to control breathing in order to accurately interpret HRV when frequency domain parameters are used (67, 68). In fact, the respiratory frequency measured in the present study ranged widely between 0.24 and 1.41 Hz, while the setting HF range for the frequency domain analysis was limited to 0.20–0.40 Hz as generally suggested for sheep and goats (2). For the HF to have a bell-shaped distribution in the frequency domain analysis, any deviation of the respiratory frequency from the HF range simply masked the effect of respiration on the frequency domain HRV parameters, particularly at lower and higher HR conditions that corresponded to lower and higher THI categories. It is though that HF is one of the major parameters of cardiac vagal activity which is strongly linked with a range of self-control such as cognitive performance, emotion and stress regulation, health, and social interactions (69, 70). However, since controlling the breathing of animals is almost impossible, the frequency domain parameters are of limited use for evaluating autonomic regulation in freely-moving animals.

In comparison with the other two domains of HRV analysis, the non-linear domain parameters (L_max_ and %DET) showed characteristic changes in the present study; they increased with the THI categories without correcting for the effect of physical activity, but only increased from 75–80 to 80 < when including the effect of physical activity. This result indicated that there was a THI threshold level of around 80 affecting the HRV parameters. Although the insusceptibility of non-linear HRV parameters to respiration is still in debate, it has been suggested that the non-linear complexity and determinism of HRV do not arise as a consequence of a respiration input into the cardiovascular oscillator (71, 72). The results of the present study suggest that, as long as animals breathe merely for gas exchange required for the metabolism, respiration does not induce non-linear HRV. However, if the breathing pattern is changed for some reason other than basal metabolism, the respiratory pattern may interfere with the central cardiovascular oscillator. Since one possible predictive marker of alterations in central autonomic regulation that may precede metabolic stress is a non-linear domain component of HRV, as suggested by Hoffmann et al. (73), we can conclude that it is beneficial to use non-linear HRV parameters as physiological heat-stress indicators.

It should be noted that the present study used only six individuals from two small ruminant species, which was one of the limitations of the present study. However, the fixed effects of THI category and species were properly analyzed with considering the effect of individuals in each species, since the effect of individual differences was treated as a random effect nested within the species in the statistical model with enough data for each animal. The results showed that the effect of species was not significant for HRV parameters except for frequency domain parameters, which indicated that the finding in particular for non-linear HRV parameters could be correctly evaluated. However, in order to strengthen the validity of the analysis and the conclusions for the effect of THI on HRV parameters and also to clarify the difference in the effect between the two small ruminant species in more detail, further studies using experimental designs with more animals would be required.

### Heat Dissipation and Heart Rate Regulation

Many of the physiological responses under heat load are evoked to maintain the core temperature constant. For this purpose, heat dissipation from animal body is promoted by vasodilation, panting, and sweating. In the present study, moderate tachycardia was observed as the THI increased, which might be in accordance with the interpretation that increases in the heart rate under heat load is evoked due to increases in blood circulation to transfer heat from the core to the periphery (34).

The initial response that maintains the core temperature under heat stress is vasodilation (74), which results from the withdrawal of sympathetic nervous activity that governs the vessel tone (sensible heat dissipation). As the heat load increases, latent heat dissipation is gradually induced. Cholinergic sympathetic activity that regulates activity of sweat glands also acts as an active local vasodilator (75) that synergistically dissipates heat. Both responses to external heat stress, vasodilation and sweating, are regulated by the thermoregulatory center in the preoptic area. However, in the case of panting, the modified breathing pattern interferes with systemic circulation, and directly changes the heart rate via cardio-pulmonary baroreceptors. This mechanism may make the regulation of heart rate more complex and chaotic.

Ambient temperature and humidity are critical factors that affect the efficacy of both sensible and latent heat dissipation, and the THI is a useful index that indicates the heat-stress level in homeothermic animals. If body temperature cannot be maintained by the responses discussed so far because of rigid heat load, heat production is suppressed by reducing feed intake, which results in reduced production (76). However, in the present study, the rectal temperatures of the animals tested did not significantly differ among the three experimental periods (Table 3). Moreover, a decrease in feed intake was not observed for the tested animals. In dairy cows, Kadzere et al. (32) reported that THI values > 78 might cause extreme distress, with lactating cows being unable to maintain their thermoregulatory mechanisms or normal body temperatures. Lemerle and Goddard (77) reported that homeostatic mechanisms could prevent a rise in rectal temperature until the THI reaches 80, which might also be applied for the small ruminants in the present study. Therefore, although some degree of disorder of thermoregulatory control shown as the change in non-linear HRV parameters might occur over 80 of THI, the tested animals could mostly cope with heat stress by promoting heat dissipation until the THI reached the threshold value.

## Conclusions

The present study revealed the effects of heat stress on HRV of sheep and goats. Under high THI conditions, HRV of the animals was decreased which might be due to the increase of sympathetic nervous system activity on heart rate regulation. From the evaluation of non-linear HRV parameters with correction for the effect of physical activity, this study could suggest the existence of a threshold value of THI around 80 for HRV. The threshold value might indicate a limit that the external stress imposes physiological non-linear heart rate regulation for the heat dissipation in order to maintain core temperature constant.

In recent years, increased interest has been paid to HRV measurement as a non-invasive technique to assess stress in animals in particular related to animal welfare. The results of the present study indicated both heat stress and physical activity levels affected HRV. Therefore, in order to investigate the effects of psychophysiological stress factors on HRV of unrestrained animals, the confounding effect of physical activity on HRV should be taken into consideration. Moreover, the use of multiple domains of HRV parameters, in particular non-linear parameters, should be recommended for investigating different characteristics of the effect of stressors on HRV.

## Data Availability Statement

The datasets generated for this study are available on request to the corresponding author.

## Ethics Statement

All of the animal experiments were approved by the Animal Experiment Committee of Kyoto University (Permit number: 27-56).

## Author Contributions

KK and KO designed the study, collected and analyzed the data, and wrote the manuscript. MM and HA collected the data and helped write the manuscript. AS and YY collected the data. YH wrote the manuscript. HK and HH helped write the manuscript. All authors discussed the results, contributed to manuscript revisions and provided approval for publication.

## Conflict of Interest

The authors declare that the research was conducted in the absence of any commercial or financial relationships that could be construed as a potential conflict of interest.
